# An exercise intervention for people with serious mental illness: Findings from a qualitative data analysis using participatory theme elicitation

**DOI:** 10.1111/hex.13141

**Published:** 2020-10-09

**Authors:** Jade Yap, Claire McCartan, Gavin Davidson, Chris White, Liam Bradley, Paul Webb, Jennifer Badham, Gavin Breslin, Paul Best

**Affiliations:** ^1^ Mental Health Foundation London UK; ^2^ Queen’s University Belfast Belfast UK; ^3^ Praxis Care Belfast UK; ^4^ Ulster University Belfast UK

**Keywords:** coproduction, SMI, exercise, participatory theme elicitation, participatory data analysis, Physical activity

## Abstract

**Background:**

People with severe mental illness (SMI) often have poorer physical health than the general population. A coproduced physical activity intervention to improve physical activity for people with SMI in Northern Ireland was evaluated by co‐researchers (researchers with lived experience of SMI) and academic researchers using a new approach to participatory data analysis called participatory theme elicitation (PTE).

**Objective:**

Co‐researchers and academic researchers analysed the data from the pilot study using PTE. This paper aimed to compare these analyses to validate the findings of the study and explore the validity of the PTE method in the context of the evaluation of a physical activity intervention for individuals with SMI.

**Results:**

There was alignment and congruence of some themes across groups. Important differences in the analyses across groups included the use of language, with the co‐researchers employing less academic and clinical language, and structure of themes generated, with the academic researchers including subthemes under some umbrella themes.

**Conclusions:**

The comparison of analyses supports the validity of the PTE approach, which is a meaningful way of involving people with lived experience in research. PTE addresses the power imbalances that are often present in the analysis process and was found to be acceptable by co‐researchers and academic researchers alike.

## 1 INTRODUCTION

Though physical activity has been found to have positive physical and mental health benefits for people with severe mental illness (SMI),^1^ exercise interventions are seldom offered as a treatment option in mental health care.^2^ Given that the estimated mortality gap for people with SMI is between 11 years^3^ and 30 years^4^ with a 20% reduction in life expectancy,^5^, ^6^ it is of public health importance to identify how to increase uptake and implementation of physical activity interventions among this population group.

People with SMI are likely to face more barriers to physical activity than the general population. These include lack of motivation, which could be due to the mental health condition itself^7^ or be a side‐effect of medication they take (eg weight gain) which may make it more difficult for people with SMI to be physically active.^8^, ^9^, ^10^ Stress, depression, disinterest in exercise, feeling unsafe or fear of injury were found to be barriers to engagement^11^ and anxiety, including social anxiety^12^ and anxiety around one's perceived exercise ability,^11^ may also prevent some people with SMI from participating in physical activity. People with mental health problems are also more likely to develop physical health conditions, such as cardiovascular disease, obesity and diabetes, than the general population, and poor physical health and tiredness resulting from these comorbidities also serve as a barrier to participating in physical activity.^11^, ^13^ Other health risk behaviours (eg cigarette consumption, hazardous alcohol use) are also more common in people with SMI and can negatively impact on a person's ability to participate in physical activity.^14^, ^15^

As well as physical activity barriers, suggestions for effective physical activity interventions for people with SMI have been proposed, with a key recommendation being that the format of exercise is structured, supervised and delivered ideally by trained fitness professionals.^16^, ^17^ A study of in‐patient nursing staff views stated the most prescribed exercise is group‐based, 3 times per week for 20 minutes.^18^ Many studies focus on the level of intensity required to benefit people with SMI, stating that physical activity should be of a moderate‐to‐vigorous intensity to have a positive effect on mental and physical health symptoms.^19^, ^20^, ^21^ In a recent scoping review, it was reported that activity can range from 30 minutes to 3 hours in order for it to reduce mental illness symptoms.^22^ Despite this growing body of evidence exploring the impact of physical activity and mental health problems, there is a lack of research using coproduction to explore the facilitators and barriers to physical activity for people with SMI. This is notable because coproductive methods have the potential to enhance the quality and relevance of research.^23^

This paper relates to the evaluation of a three‐month physical activity programme for people with SMI in Northern Ireland that took place in 2019. A team of lived experienced researchers with an SMI were employed to work on the study. Through adopting a qualitative, participatory approach, the study aimed to increase knowledge on what works to engage people with SMI in sustained physical activity; explore current barriers and facilitators to physical activity; and provide practical solutions to inform delivery of services in Northern Ireland. Given the emphasis on the study's coproductive approach, it was important that people with lived experience of mental health problems participate in the analysis. The initial term that was used in the recruitment process to describe the researchers with lived experience of SMI was ‘peer researchers’; however, this was later changed, as the peer researchers questioned the definition during the capacity building process of the PTE approach, and concluded that the term ‘co‐researchers’ was more in line with the spirit of coproduction and equality; thus, this is the preferred term in this study. Both the co‐researchers and the academic researchers participated in the analysis process, with the former able to draw on their lived experience when interpreting the data.^24^, ^25^

In practice, coproduction is more common at the initial planning and design stages of a study, whereas the analysis and report write‐up stages tend to be dominated by academic researchers,^26^, ^27^ perhaps due to issues around time and cost. Tensions may also arise when experts‐by‐experience and/or professionals work together,^28^ particularly during these later stages which are sometimes conceptualized as more formal and distinct to the earlier research stages.^29^ Indeed, academic researchers may consider data analysis to be one of their key skills and therefore be reticent to share power with co‐researchers at this stage.^30^, ^31^

To ensure that the project was fully coproduced, the data analysis approach adopted for this study was participatory theme elicitation (PTE), which has been effective in other research projects involving co‐researchers.^32^, ^33^ Whilst many other participatory data analysis methods focus on coding,^34^, ^35^, ^36^ this may result in an imbalanced analysis process that is disproportionately influenced by academic researchers.^37^ PTE, which is a five‐step process (that consists of data selection, capacity building, open sorting, data grouping, data analysis and interpretation), builds on common participatory methods centred around coding but uses network analysis techniques to facilitate generation of themes. Applying this to health and social sciences research, quotes from interviews or focus groups are included on the cards for co‐researchers to sort. Sifting through large volumes of raw data for analysis can be complex and time‐consuming, so it is often a barrier to participation; however, sorting through cards with a smaller number of key phrases or quotes is more manageable.^38^ Following this, network analysis methods are used to determine sorting patterns across all researchers to inform the selection of the final themes. This innovative approach serves to address power imbalances and democratize the process, as the independence of the network analysis results reflecting everyone's independent sorting process serves to minimize the influence of the academic researchers in the analysis process.

The key aim of the paper was to compare analyses from both co‐researchers and academic researchers to validate the findings of the study and explore the validity of the PTE method in the context of the evaluation of a physical activity intervention for individuals with severe and enduring mental health problems.

## 2 METHODS

Ethical approval for the exercise intervention was granted by The School of Social Sciences, Education and Social Work Ethics Committee, Queen's University Belfast, on 5 July 2018. Details about the methods of the study are outlined elsewhere^39^; this section relates to the PTE approach employed for data analysis only.

### 2.1 Recruitment

The four co‐researchers (two males and two females between 30 and 59 years old) who participated in the two‐day analysis workshop were recruited previously to work on the pilot study and had contributed to the programme design and data collection. All participants had obtained secondary‐level education and two held university degrees.

There were four academic researchers (two males and two females between 34 and 54). One was the key researcher on the project; another was a mental health researcher; another was a key partner on the project; and the remaining researcher acted in an advisory capacity on the project. All participants held university degrees and post‐graduate qualifications, ranging from master's degrees to PhDs.

PTE consists of five steps (presented below), which is discussed in greater depth by Best and colleagues (2017) and involves (a) data selection, (b) capacity building, (c) open sorting, (d) data grouping and (e) data analysis and interpretation.

#### 2.1.1 Step 1: Data selection

Two individuals from a partner organisation, who were not previously involved in data collection, independently reviewed the transcripts of six focus groups and selected standalone representative quotes. More than one person is needed to select quotes, and for practical purposes (relating to time and resources), two individuals participated in data selection. It is usually recommended that one of the quote selectors has lived experience. In this instance, one individual had academic research experience and was part of the Management Steering Group Committee of the study; the other had research expertise stemming from their lived experience and was head of the study's Advisory Group. The two individuals were chosen as they were not involved again in the process until Step 5 (Data Analysis and Interpretation) which is important to ensure that no single member of the research team is involved at every stage, thus limiting their ability to influence the process.

After selecting quotes individually, the two then met to agree on a final list of 89 anonymized quotes (ID01‐ID89). While higher than the number of quotes used in previous PTE studies,^32^, ^33^ it was felt that these quotes accurately maintained the essence of the focus group conversations and provided a natural opportunity to explore the acceptability of PTE with more quotes.

#### 2.1.2 Step 2: capacity building

Given that the co‐researchers had already worked on the project, they were well‐versed in the details of the physical activity intervention and evaluation. In other cases, co‐researchers should receive an overview of the intervention and research project at this stage. Researchers were provided with a slide which contained instructions for the sorting task they would undertake that would later help them to develop themes (see Appendix A).

#### 2.1.3 Step 3: open sorting

In separate sessions, both groups of researchers were presented with information packs which included the 89 quotes, each individually cut out; and a consent form and blank sheets of paper to create labels. An evaluation form (see Appendix B) was also included for participants to complete at the end of the task. Researchers would typically also be provided with a project information sheet; however, in this case, the researchers were already aware of the background to the study as well as its aims and objectives due to their prior involvement in the study. They were, however, not aware specifically of the questions that preceded each of the responses selected as quotations as one of the main pieces of guidance for quote selection was that they could be easily understood as standalone statements.

They were given instructions on a PowerPoint slide (see Appendix A) that remained visible throughout, which instructed them to sort the quotes into piles based on similarity, using whatever criteria they found relevant.^40^ There had to be at least two piles and no ‘miscellaneous’ pile, and each researcher labelled and stapled their piles of quotes. The sorting process was undertaken independently so that researchers were not able to influence or be influenced by each other. Two facilitators were present to answer questions but did not offer opinions or interpretation.

#### 2.1.4 Step 4: data grouping

The piles of data of both researcher groups were inputted into an Excel spreadsheet by PB and CM (who were not involved in the original selection of quotes). The spreadsheet contained three columns: (1) the anonymized identifier of the researcher; (2) the ID quote; and (3) the label (in this case, numerical) representing the pile. It was uploaded to a previously developed user‐friendly web‐based application which conducted network analyses and produced a downloadable network diagram. The analysis also applied the Louvain community detection algorithm to assign themes that reflect the combined sorting patterns for each set of researchers.^41^

At the end of this step, each group had two documents: i) one network diagram each and ii) a sheet with the 89 quotes separated into the groupings identified in their respective network diagram.

#### 2.1.5 Step 5: data analysis and interpretation

On day 2 of the workshop, both groups of researchers were presented with their respective network diagram and the sheet of quotes, which was used as a basis for discussion to generate themes in each group. Two facilitators (one for the co‐researchers and another for the academic researchers) recorded themes and initial codes on flipchart paper. The two groups then came together to compare and agree the final list of themes, which are summarized in Appendix C.

## 3 FINDINGS

### 3.1 Network analysis

**FIGURE 1:**
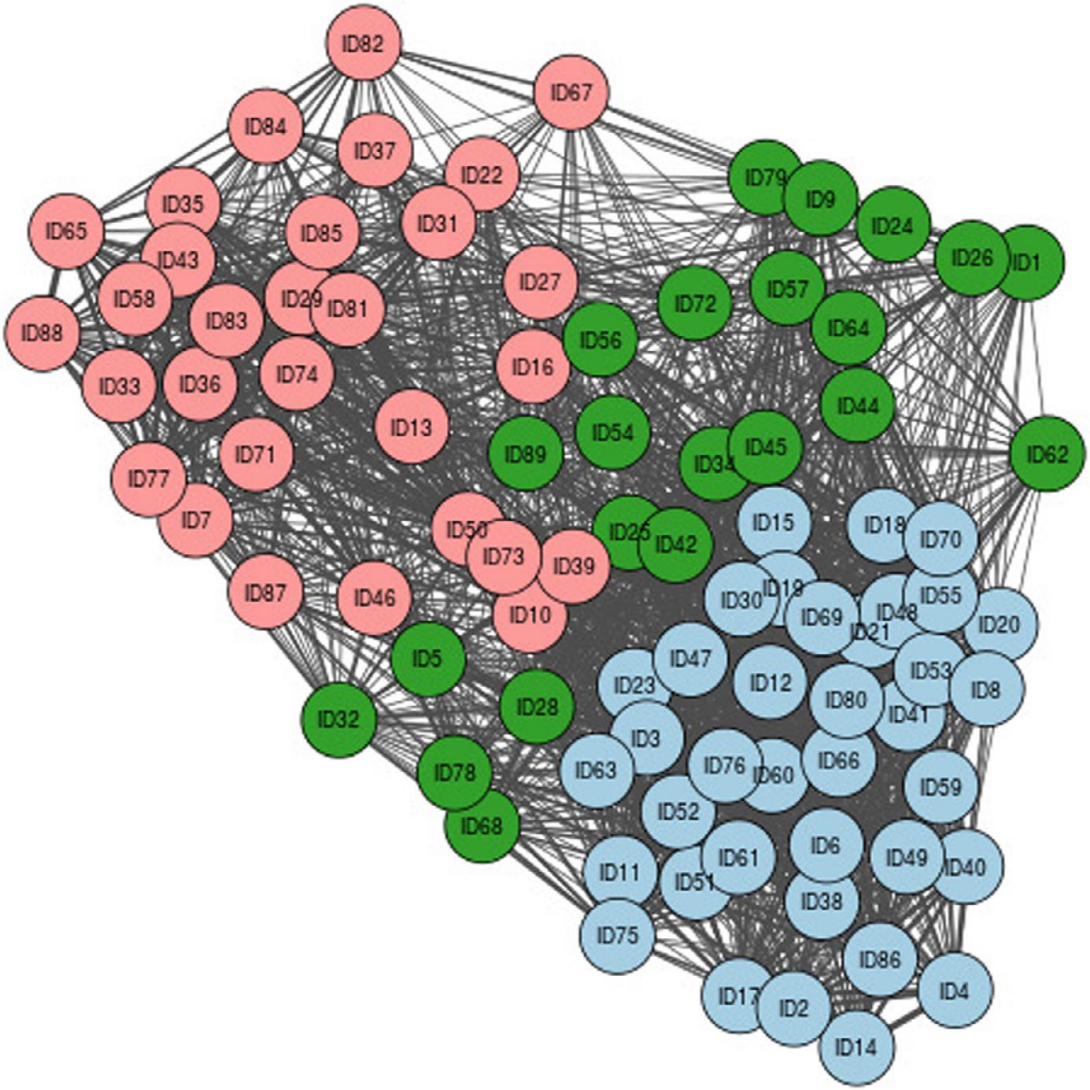
Network diagram and sorting results for co‐researchers. The co‐researchers categorized the quotes into three main groups represented by the three different colours. Group 1 is represented as pink; group 2 is green; and group 3 is blue

**FIGURE 2:**
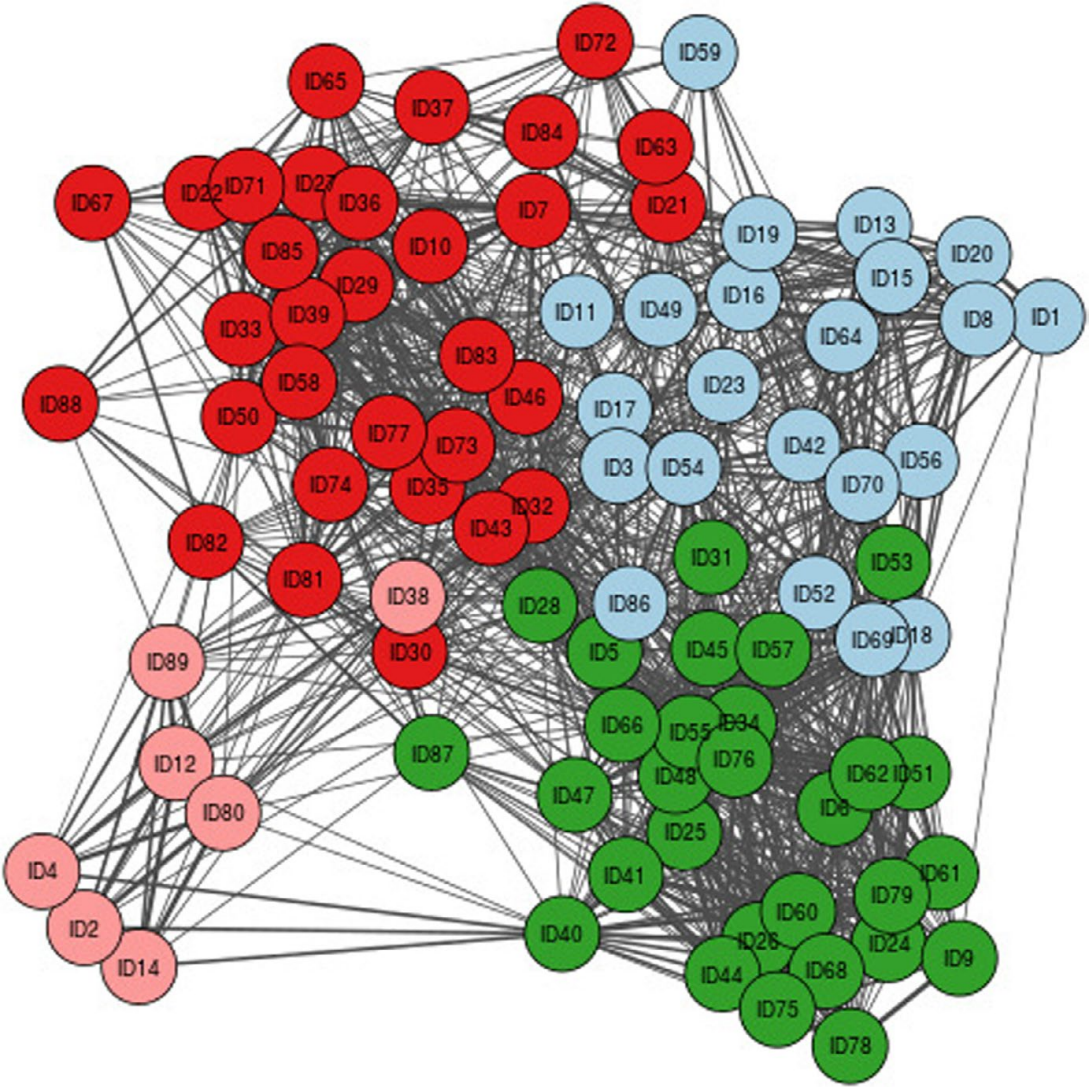
Network diagram and sorting results for academic researchers. The academic researchers categorized the quotes into four main groups represented by the four different colours. Group 1 is represented as red; group 2 is pink; group 3 is blue; and group 4 is green

In the co‐researchers’ and academic researchers’ network diagrams (Figures 1 and 2), each coloured circle represents a node. Pairs of nodes are connected if at least one person placed the same two quotes in the same pile. The thicker the line, the greater the number of researchers that have grouped those quotes into the same pile. The different colours in the diagrams represent the different groupings found by the Louvain algorithm.

Both groups were informed that information regarding the strength of relationship between quotes and groups could be gleaned visually from the network diagrams by looking at the proximity of nodes to each other and the thickness of lines connecting them together.

### 3.2 Theme generation

The co‐researchers’ PTE analysis identified three unique groupings (Figure 1), whilst the academic researchers’ analysis identified four (Figure 2). The quotes grouped by colour from the two network diagrams are listed in Appendix D.

Figure 1 was presented to the co‐researchers; Figure 2 was presented to the academic researchers. Both groups met separately on day 2 of the workshop to review the groupings identified in the diagram and ascertain themes. The facilitator of each session emphasized that they were not bound to the groupings in the diagram; the network diagram was simply there to stimulate discussion and perhaps add greater depth and detail to their discussion.

After this session, the co‐researchers and academic researchers met and the two facilitators presented the themes identified by the group they had just facilitated. This encouraged a wider discussion among both groups who reflected on similarities and differences across groups, and the final theme list was developed. For this paper, the list of themes from the two‐day PTE session is of interest, though final themes in the project report differ slightly as co‐researchers and academic researchers met later to reflect on the draft ‘Results’ section of the report to ensure that it accurately conveyed the themes they had discussed.^39^

### 3.3 Comparison across co‐researchers and academic researchers

The analyses of the co‐researchers and the academic researchers are presented in Tables 1 and 2, respectively. In the co‐researchers’ network diagram, there were three coloured groupings and thematic relationships were developed within all three (step 4); in the academic researchers’ network diagram, there were four coloured groupings and thematic relationships were developed within all four. A comparison of the two groups’ analyses is explored in this section.

**TABLE 1:** Themes generated by co‐researchers using the PTE approach

Theme	Quote IDs	Dominant colour grouping	Percentage of quotes in theme belonging to same dominant colour grouping
1. Social element/ group dynamic	14, 4, 2, 40, 49, 12, 80, 89	Blue	100%
2. Preconceptions and barriers to exercise	10, 36, 16, 46, 37, 7, 77, 74, 43, 29, 27, 81, 88, 65, 84	Pink	100%
3. Approach from trainers to participants	13, 87, 7, 58	Pink	100%
4. Practical issues	89, 22, 11, 64, 3, 54, 42, 59, 10, 71	No dominant colour grouping—4 green; 4 pink; 3 blue	N/A
5. Education/ knowledge and awareness	78, 24, 28, 57, 9, 32, 5, 42, 1, 62, 44, 45, 25, 54, 34, 26, 68	Green	100%
6. Personal responsibility	32, 30, 83, 47, 15, 40, 69, 41, 23, 17, 19	Blue	82%
7. Change in behaviour and perspectives	ID17, ID66, ID70, ID48, ID32, ID35, ID23	Blue	71%

**TABLE 2:** Themes generated by academic researchers using the PTE approach

Theme	Quote IDs	Dominant colour grouping	Percentage of quotes in theme belonging to same dominant colour grouping
1. A social environment	2, 4, 12, 14, 80, 89, 10	Pink	85.7%
2. Unique qualities of trainers/ programme	13, 15, 18, 20, 56, 19, 8	Blue	100%
3. Agency	23, 49, 54	Blue	100%
4. Physical adjustments to programme	3, 11, 17, 52, 69, 70	Blue	100%
5. Benefits of physical activity			
a. Physical benefits	a. 61, 45, 51, 66, 79, 24, 62	a. Green	a. 100%
b. Physical and mental benefits	b. 6, 75, 78	b. Green	b. 100%
c. Mental benefits	c. 5, 9, 26, 25, 34, 41, 44, 55, 57, 47, 60, 68, 76	c. Green	c. 100%
6. Barriers			
a. The 'gym' environment	a. 46, 21, 65, 73, 84, 85	a. Red	a. 100%
b. Barriers more generally for participants	b. 36, 50, 33, 74, 37, 77, 27	b. Red	b. 100%
c. Practical, process‐related barriers	c. 63, 67, 71, 72, 22, 10	c. Red	c. 100%
7. Long‐term habits	29, 32, 35, 39	Red	100%

Both groups included a theme on the importance of the social aspect of the programme with similar labels to convey this—‘social/group dynamic’ and ‘a social environment.’ The convergence of these themes across both groups is evident by the choice of quotes they selected to develop this theme, such as:

'But there's very much a community spirit already established with them, but it's carried into our groups. It makes our role with them so much easier.' [ID4]

Barriers to engaging in physical activity were also explored by both groups; however, both groups adopted a slightly different focus which complemented each other and provided a deeper understanding of the data. The co‐researchers focused on exploring how preconceptions people with mental health problems may have about themselves, such as feeling unable to cope with the pace of physical activity and being worried about other people's perceptions, could serve as a barrier to engaging in exercise. In contrast, the academic researchers listed ‘barriers’ as a broad umbrella theme, with three subthemes that elaborated on different aspects of barriers. Despite these different perspectives, thematic alignment is evident as there is overlap between the co‐researchers’ barriers theme and the academic researchers’ subthemes. For instance, with the academic researchers’ first subtheme—‘the “gym” environment’—some of the same quotes highlighting participants’ negative perceptions about their abilities, which they often spoke about in relation to the gym, appeared in both groups to explore similar ideas such as:

'I couldn't possible go to a gym, I would be sick… I just couldn't exert myself like that, I’d have no energy, nothing…’ [ID46]

There was also overlap between the co‐researchers’ barriers theme and the academic researchers’ subtheme of ‘barriers for participants more generally’ which explored, among other things, other people's perceptions and feeling too old for certain types of exercises.

The final barriers subtheme identified by the academic researchers related to the practical, process‐related obstacles in the project. The co‐researchers similarly explored this in a separate theme which they labelled ‘Practical issues’ which discussed not only barriers such as the challenge of getting GP approval but also more generally practical aspects of the programme, such as the ideal size of a group and the considerations that the trainers needed to take into account when delivering a programme to people with severe and enduring mental health problems.

Whilst both groups had a theme on the trainers of the programme, the co‐researchers focused on the trainers’ approach, whereas the academic researchers explored the unique qualities and skills that trainers needed for this programme to be effective. Similarly, though the themes of ‘personal responsibility’ (co‐researchers) and ‘agency’ (academic researchers) appear quite aligned, with each referring to a growing sense of empowerment among participants, there is little convergence across groups in relation to the selection of quotes, indicating that there are differences in what the themes are exploring stemming from the researchers’ varying, though complementary, perspectives. The co‐researchers’ theme of personal responsibility appeared to have a slightly broader focus that extended beyond physical activity, with one quote in this category discussing how the participant was now thinking more carefully about their dietary choices [ID47]. There was also recognition that in order for participants to take personal responsibility for their behaviours, extra support may be required to ensure people feel more confident and empowered about their choices. Indeed, the co‐researchers discussed that though everyone was working on something individually they all appeared to support each other collectively as a group [ID15]. In contrast, the academic researchers’ ‘agency’ theme was focused more on the participants feeling more empowered and able to take control of their physical activity behaviours.

This is a similar case with the themes of ‘change in behaviour/ perspective’ (co‐researchers) and ‘long‐term habits’ (academic researchers). In the former, the co‐researchers discussed that there is a clear link between the participant recognising personal responsibility for their health and then altering behaviours in accordance with that [ID45], and a sense that participants’ attitudes around engaging in physical activity were broadening, resulting in a changed perspective towards physical activity [ID70] which encouraged them to try out different things that they would not have previously, such as park outdoor machines [ID17]. The academic researchers’ ‘long‐term habits’ theme had a narrower focus and consisted of quotes that referred to the long‐term habits that impact physical health, including eating habits [ID29, ID32], smoking [ID35] and decreased daily exercise with age [ID39].

Another key difference relates to the language used across groups, with the co‐researchers’ themes employing less academic and clinical language in comparison with the academic researchers. One theme identified by the academic researchers was ‘agency’, which is an academic psychological concept that refers to the degree with which an individual feels they have control over actions and consequences.^42^ The corresponding theme identified by the co‐researchers used the non‐academic term of ‘personal responsibility.’ This difference in language might be an important consideration in how to engage with people with SMI to promote change. Another difference across the groups related to the structure of the theme list; whilst the co‐researchers listed seven standalone themes, the academic researchers included subthemes under two umbrella themes. This is likely to be due to the academic researchers’ experience of undertaking thematic analysis and their familiarity with various formats of categorising qualitative data.

The thematic alignment and congruence of some themes across groups support the validity of the PTE approach. There are also important differences across groups, reflecting each group's different, equally valuable perspective, which suggests that PTE is a valuable methodology that could improve sensitivity to additional findings.

At the end of the two‐day workshop, the two groups of researchers convened to discuss and compare the themes each group had identified using PTE and ascertained a final list of themes. While the focus of this paper was to compare the themes generated across groups, the final list of themes from the workshop is included in Appendix C, which serves to demonstrate how PTE plays an integral role in the overall analysis approach.

## 4 DISCUSSION

This paper sought to strengthen the validity of the findings of an evaluation of a physical activity intervention for people with SMI in Northern Ireland; and explore the validity of the participatory theme elicitation by comparing analyses between lay and academic researchers.

### 4.1 Summary of key findings

Though people with SMI are disproportionately excluded from physical activity, our qualitative findings highlight that they can enjoy physical and mental health benefits, including improved sleep and increased energy, from a physical activity intervention that is of a lower intensity than those often recommended in literature.^19^, ^43^

The data analysis identified facilitators to engaging people with SMI in physical activity, including focusing on the social component of the physical activity programme, which is discussed in prior research.^44^, ^45^ The social aspect of this intervention enhanced participants’ motivation to continue attending and engaging in the programme and fostered a sense of community among the group. The analysis also highlighted the value in tailoring the intervention to the needs of the participants as a way to keep participants motivated and engaged, which is also supported in previous literature.^46^, ^47^ This is promising as it means that even low‐intensity interventions can be engaging and beneficial for people with SMI, especially those with lower levels of confidence and ability.

The findings of the study confirm the various barriers that people with SMI face to engaging with physical activity that are well‐outlined in the literature, including lack of motivation, feelings of self‐consciousness around one's own appearance or being in the gym environment.^7^, ^13^ Side‐effects of medication, symptoms of one's mental health condition, lack of access to equipment and lack of time were also raised as barriers by participants, which correspond with findings in the literature.^7^, ^11^ However, for this pilot study gaining GP approval was a key barrier, which is not often discussed in the literature and is an important logistical consideration when designing a physical activity programme.

Though there are indications in the literature that people with SMI may be less knowledgeable about the mental health benefits of physical activity,^48^ the findings of this study suggested a growing awareness among participants of this. Given the aforementioned delays in getting GP approval, it may be valuable to encourage and support GPs (and other health professionals) in increasing awareness of the mental health benefits (in addition to the physical health benefits) of exercise to allow them to promote physical activity to their patients with SMI with greater confidence.^49^, ^50^

### 4.2 A co‐researcher's reflection on PTE

One of the co‐researchers reflected on using PTE in the data analysis, highlighting their initial uncertainty around the methodology which soon paved the way to an understanding of its benefits and its innovative approach. The co‐researcher also explored how their own lived experience of SMI impacted the project:

'As the study period of 12 weeks progressed, I was wondering, how is any data that we produce going to be collated and presented, unless we were going to take biometric measurements from everyone at the end? When I discovered we would be using the quotes from discussions with the participants I was still confused.

The PTE was a revelation in that randomly generated qualitative data could be processed and presented as a quantitative display.

More generally I felt that with having lived experience of mental ill health (which led me to gain knowledge working with AWARE and the Recovery Colleges as part of my own recovery) I was confident and empathetic in working with our study volunteers and participation in the study further enhanced my understanding of mental ill health in the community.'

### 4.3 The role of the facilitator

The facilitator was important in encouraging discussion among the co‐researchers and developing the final list of themes. In this study, the key facilitator was a lived experience researcher (CW) with extensive experience in facilitating workshops with people with mental health problems, which helped to reduce any power imbalance and promote joint decision making. He had also been involved in the project from its inception and selected with another researcher the 89 quotes (step 1). This familiarity with the project and the PTE methodology was invaluable in building a rapport with the co‐researchers and relaying the methodology to them in an accessible and engaging way.

### 4.4 Acceptability of PTE

Feedback on the methodology from the academic and co‐researchers indicates that PTE is a valuable method of engaging people meaningfully in research. From a discussion at the end of the workshop between the academic researchers (excluding Paul Best who developed PTE) and the co‐researchers, it was evident that both found it to be an acceptable participatory data analysis method. The academic researchers reflected that despite initial concerns, they found it to be a useful way of engaging people, with a key strength being the independence of the network analysis results which play a key role in levelling the power relations in participatory data analysis. Despite support for the acceptability of PTE, this approach should not be considered to be the ‘only’ way to explore data but can instead be viewed as a useful tool in a sequence of analysis.

The co‐researchers found PTE to be acceptable and ‘enjoyable’ and were able to pick up the process relatively quickly. One lay researcher stated that they were surprised by ‘how much information was received by random snippets of people's thoughts,’ emphasising the value in the approach and the insights it garnered. Regarding the number of quotes in the study, one lay researcher felt that this was ‘just right’, while another commented that ‘you could easily handle more than 89.’ The selected 89 quotes were described as ‘easy to understand’ and categorize.

### 4.5 Strengths and limitations

Incorporating co‐researchers in the analysis strengthened the project findings by providing an insider perspective. The same four co‐researchers were present for both days of the workshop which allowed for consistency during the process. The two people who selected the 89 quotes from the transcripts were not involved in the initial data collection, thus minimising the potential for selection bias. Though the number of quotes (89) was found to be appropriate, it is still the case that a relatively small number of quotes must be selected to ensure that the process is manageable in the given time period. This selection process may therefore be an important limitation of the approach and create another potential source of bias.

Despite the intentions of PTE to minimize researcher influence and democratize the analysis process, its requirement that co‐researchers undergo training means that it is subject to the ‘professionalisation paradox’^51^ criticism, which refers to the fact that co‐researchers will necessarily undergo some degree of professional socialisation, thus limiting the unique value of 'layness’ on the research. It could be argued, however, that developing research methods, knowledge and skills does not necessarily diminish the contribution of lived experience. It is possible that people can be both experts‐by‐experience and experts‐by‐research method training. In addition, the training component of PTE is purposely short compared to other qualitative data analysis methods and, as the co‐researchers in this study attested, relatively straightforward to pick up.

### 4.6 Conclusion

The qualitative analysis highlighted that people with SMI can enjoy mental health and physical health benefits from engaging in physical activity, even at low levels of intensity. Key facilitators and barriers were identified, many of which mirrored findings in the literature; however, some unique insights were garnered, including the challenge in gaining GP approval for patients with SMI to engage in physical activity programmes.

This paper compared themes generated by co‐researchers and academic researchers in an evaluation of a physical activity programme, and found thematic alignment and congruence, which supports the validity of the PTE approach. PTE was also found to be a beneficial way of involving people with lived experience in research without having them go through a large amount of training. The approach also allowed for differences between the analyses conducted by the co‐researchers and academic researchers to emerge, which may not occur in standard data analysis, and was able to balance out some of the bias towards the type of information that might be seen as important from each group. The methodology created some distance in the analysis process between the academic team which helps to minimize scientific researcher input/influence. Involving people with lived experience of mental health problems as co‐researchers in the analysis provided a unique lived experience perspective that strengthened and enhanced the findings. The co‐researchers found PTE to be acceptable even with a larger number of quotes than previous studies using the PTE approach. Future work using the PTE approach would benefit from trialling a larger number of quotes to determine at what point acceptability ceases.

## CONFLICTS OF INTEREST

Professor Gavin Davidson is Praxis Chair of Social Care at Queen's University Belfast, and this post is partially funded by Praxis Care. He is also on the Development Committee of the Mental Health Foundation.

## AUTHORS’ CONTRIBUTION

PB had the original idea for the paper and had oversight of the research project. The data analysis workshops were set up by PB, CM, GD and PW; the co‐researcher and traditional researcher sessions were facilitated by JY and CW, supported by GB. JB conducted the network analysis and advised on methodological aspects of the project along with PB. Comparison of the themes across workshops was undertaken by JY, CM and CW. JY wrote the first draft of the paper, of which LB wrote a section, and all the authors edited and approved the paper.

## Data Availability

The data that support the findings of this study are available in the supplementary material of this article.

## APPENDIX A

Instructions that were shown on a PowerPoint slide during the sorting task:

○ We would like you to group similar quotes together
○ These can be made into smaller piles
○ Each pile must have more than one quote
○ This is not a test and there are no right or wrong answers!
○ We need you to work alone
○ Please label piles
○ Please ask us if you don't understand the quote

## APPENDIX B Participatory Theme Elicitation (PTE) Training Evaluation Form

We would appreciate if you could take a few minutes to share your opinions with us so we can improve on this training.

## APPENDIX C

Final list of themes agreed on by co‐researchers and academic researchers at the end of the two‐day workshop:

1. Social aspect
2. Barriers to physical activity
3. Practical issues
4. Approach from trainers to participants
5. Education and awareness
6. Personal responsibility
7. Behaviour/perspective change
8. Life‐long habits

## APPENDIX D

### CO‐RESEARCHER PTE NETWORK ANALYSIS GROUPINGS

**Group 1: Blue**

**Table:**

No	Quote
4	But there's very much a community spirit already established with them, but it's carried into our groups. It makes our role with them so much easier.
14	It was already a pre‐made social group, they did meet up once a week already…
2	Community inside and outside of the gym, to incorporate that mental health element into it
17	When we were out in the park, you know the way you see now these machines that are out in the park anyway, so I was able to get them using things like that.
86	And see that leisure centre, the people there, they are just fantastic. You are welcome in straight away and when you do the exercise you get coffee, biscuits and tea.
40	I would take the dogs out for a walk… I enjoy that because you actually meet people and have a wee natter and it's good to talk
49	Maybe we could meet and go for a wee walk or something ourselves? We could meet at the park or something even? That would be good, walk round the park
38	Walking groups is something I would be very keen on.
59	And the Recovery College, that's another door and for them to bringing it on to their prospectus
8	We knew, they felt comfortable because I explained my……I was very open and transparent about my own issues and what have you
6	The progression over the 12 weeks was phenomenal.
11	The background work and the background checks, which [name removed] and [name removed] had done, had all been put in place. So really that was the hard, mundane bit. The easy bit for us was delivering, because all that was done.
19	You're here and that's it, that's all you have to do, just come and we'll scale everything right back.
75	It just means good for your bones and your brain…it stimulates your mind and you get a laugh from it.
52	See I was able to keep up more with chair exercise than what I was with walking, so I was
61	It makes ya sleep better as well. I’ve been sleeping a lot better.
20	It's actually caring about the individual that you're training.
3	I think it's more the environment with mental health, than I think as it's what time of day are you taking them in at, is it busy, are you indoors or are you outdoors, who else is around, music wise is it quiet…..?
21	Educating, if that's the right word, but making them aware that the word exercise doesn't necessarily have to be associated in a gym environment, it could be something else.
12	There has to be a balance, very much in terms of physical and social aspect of it and the group size is pretty important
51	I haven't really tried, tested my weight but I have lost inches
66	I think it got a bit easier every week. The more we did, I mean, I was feeling a lot fitter in the last few weeks of it.
70	Although < name removed > wasn't keen on the resistance bands, he bought two dumb bells, he uses the dumb bells.
53	Them Fitbits are popular too, I would love one, so I would!
48	Whereas in the house you'd be saying do this or I’ll just lie down or I’ll just sit here but you knew you were coming here so you picked yourself up to come
41	Definitely being outside, definitely clears your head
80	When you are in a group, you have more motivation because everybody around you is doing it.
60	I enjoy it. It lifts your mood a bit…
69	He got us to walk up the hill, you know, so we got a bit of, er, more benefit out of walking up a hill and he got us to kind of walk a bit further each week
23	Momentum has started now, so it needs to keep going.
63	I think, for it to have a proper long…you would need to be doing it over more than ten or twelve weeks.
15	That need that extra support, need that bit more encouragement, that needed more accountability and they did need more guidance on how to perform exercises.
47	It's to think about getting yourself that bit of time for you, as well so it is…to give yourself time to get your head clear and whatever else
30	You have to have the willpower, you have to have the determination, you have to have the stamina and you have to have the strength to say no.
18	Patience, empathy, that was one of the big things we kind of picked up on
76	You feel happier, more motivated, you get more motivated as the weeks go on you know.
55	I think for all of us, it's got us motivated to feel a lot better about ourselves

### ACADEMIC RESEARCHER PTE NETWORK ANALYSIS GROUPINGS

**Group 1: Red**

**Table:**

No	Quote
88	There's a friend of mine's going to Slimming World and she lost eight pounds in the first week and it must have been all fluid because the second week, she gained two and she had stopped her diet.
67	She was waiting on a letter to come from the doctor. They did say she was alright but the letter didn't come through in time before the course starting so she missed out.
22	GP referrals are so far behind over here, compared to the rest of the UK
71	There was < name removed > wanted to do it but she asked to late. She asked too late, she asked two or three weeks in and er she was told she couldn't do it because it would take too long to get clearance for it.
27	People think because you've got a weight problem, you can't exercise but that's a stereotype.
36	Too much, eh…, too much effort and exertion, I’d be exhausted, I just couldn't cope.
37	Exercise would probably have put me off too.
65	If you are going to go to the gym you feel self‐conscious about your weight so it would deter ya from actually goin’.
16	In my own example for me when I was getting into this, because of my own anxiety and depression stuff, I used the think, ‘Jeepers do I look like a personal trainer?’. I had to question myself saying, what does a personal trainer look like?
29	But you go into denial I think when you're kind of eating and eating and comfort eating sometimes if you're not feeling mentally well.
85	I would love to try the treadmill but we'd end up having to go to the leisure centre, get involved and talk to somebody.
39	As a child I would've been very active and even as an adult, I would have walked a couple of miles a day and then…health just, you know…
33	Well, that the thing if it was raining I wouldn't go out like, you know. If it was raining I’d stay indoors.
58	It's a generational thing of trauma, that's another big issue that comes with living here, hopefully it gets better
50	My daughter goes to yoga and she's trying to encourage me to go and I say I’m too old for that
82	One physio told me to do it and one physio told me not to so….
74	I’ve just got a lazy streak at the minute, I haven't done much recently.
77	You sit about the house, you get into a habit, a bad habit.
73	Yea, well, I’d been a member of the gym before but I joined again – although I haven't been.
35	I’ve been smoking for umpteen years and it's hard not to be going into the shop and buying a packet of cigarettes. It's hard to do that like, you know?
81	If you are doing it on your own, it's easy to stop.
43	I’m not a very good sleeper
32	Now I will admit to it I was eating sweets, chips, chocolates, everything. I had no respect or regard for my health. For some reason something's just clicked in my head…
46	I couldn't possibly go to a gym, I would be sick…I just couldn't exert myself like that, I’d have no energy, nothing, but they're very good, they're very good…
83	When I took it on and I decided that I was going to do this, I didn't think it was going to be as hard.
30	You have to have the willpower, you have to have the determination, you have to have the stamina and you have to have the strength to say no.
84	Well, anytime I think of exercise I think of somebody sweating and getting really really fit.
7	It was a very different type of clientele than what you would generally work with. It was obviously more severe. As [name removed] said I did find it was hard to keep them motivated and keep them committed to it.
72	Er, erm, may be tell people at the start what's involved more you know, what type of exercises, we didn't really know.
63	I think, for it to have a proper long…you would need to be doing it over more than ten or twelve weeks.
21	Educating, if that's the right word, but making them aware that the word exercise doesn't necessarily have to be associated in a gym environment, it could be something else.

**Group 2: Pink**

**Table:**

38	Walking groups is something I would be very keen on.
89	I think that as long as we keep this size. Becomes too big and it scares people.
12	There has to be a balance, very much in terms of physical and social aspect of it and the group size is pretty important
80	When you are in a group, you have more motivation because everybody around you is doing it.
4	But there's very much a community spirit already established with them, but it's carried into our groups. It makes our role with them so much easier.
2	Community inside and outside of the gym, to incorporate that mental health element into it
14	It was already a pre‐made social group, they did meet up once a week already…

**Group 3: Blue**

**Table:**

19	You're here and that's it, that's all you have to do, just come and we'll scale everything right back.
59	And the Recovery College, that's another door and for them to bringing it on to their prospectus
16	In my own example for me when I was getting into this, because of my own anxiety and depression stuff, I used the think, ‘Jeepers do I look like a personal trainer?’. I had to question myself saying, what does a personal trainer look like?
49	Maybe we could meet and go for a wee walk or something ourselves? We could meet at the park or something even? That would be good, walk round the park
11	The background work and the background checks, which [name removed] and [name removed] had done, had all been put in place. So really that was the hard, mundane bit. The easy bit for us was delivering, because all that was done.
13	They all had completely different interests and backgrounds and that was challenging…
15	That need that extra support, need that bit more encouragement, that needed more accountability and they did need more guidance on how to perform exercises.
20	It's actually caring about the individual that you're training.
8	We knew, they felt comfortable because I explained my……I was very open and transparent about my own issues and what have you
1	We are actively involved in coaching clients that have a mental condition of some shape or form, whether it be an official diagnosis or not.
64	All the exercises we did with him, he, brought sheets with him and he photocopied them into a booklet for us.
42	I was very, very grateful for the pre‐assessment because unknown to me I had high blood pressure and had no idea
70	Although < name removed > wasn't keen on the resistance bands, he bought two dumb bells, he uses the dumb bells.
56	They're not turning around going like putting you down in front of anybody
52	See I was able to keep up more with chair exercise than what I was with walking, so I was
69	He got us to walk up the hill, you know, so we got a bit of, er, more benefit out of walking up a hill and he got us to kind of walk a bit further each week
18	Patience, empathy, that was one of the big things we kind of picked up on
54	We're just disappointed, whenever the weeks we couldn't come, you know?
86	And see that leisure centre, the people there, they are just fantastic. You are welcome in straight away and when you do the exercise you get coffee, biscuits and tea.
3	I think it's more the environment with mental health, than I think as it's what time of day are you taking them in at, is it busy, are you indoors or are you outdoors, who else is around, music wise is it quiet…..?
17	When we were out in the park, you know the way you see now these machines that are out in the park anyway, so I was able to get them using things like that.
23	Momentum has started now, so it needs to keep going.

**Group 3: Green**

**Table:**

53	Them Fitbits are popular too, I would love one, so I would!
31	I think exercise is very good, I do a lot of walking but I don't do, erm, I don't do exercise at all, like you know?
45	I have underactive thyroid, I have everything that goes against you for energy and I would have just been in bed and I just had to push myself to come here and I feel a lot better, I definitely, definitely do
57	And you know with us having mental health issues, we see the bigger picture…
28	OK I am limited with the weight but I’m willing to try, I’m willing to do my best that I can do, you know? So, exercise for me is everything.
5	For a guy who was not getting gup until half one, two o'clock every day, because he just didn't see the point in doing it, to getting up every morning, getting himself into a routine…
66	I think it got a bit easier every week. The more we did, I mean, I was feeling a lot fitter in the last few weeks of it.
55	I think for all of us, it's got us motivated to feel a lot better about ourselves
76	You feel happier, more motivated, you get more motivated as the weeks go on you know.
34	My mental attitude will be different and I’ll feel more in the frame of mind to get on with life and enjoy it.
48	Whereas in the house you'd be saying do this or I’ll just lie down or I’ll just sit here but you knew you were coming here so you picked yourself up to come
25	Erm, you realise you can get out of a rut when you're exercising, you don't feel you know you're stuck in a rut, you can change the way you feel.
41	Definitely being outside, definitely clears your head
47	It's to think about getting yourself that bit of time for you, as well so it is…to give yourself time to get your head clear and whatever else
40	I would take the dogs out for a walk… I enjoy that because you actually meet people and have a wee natter and it's good to talk
87	I know a fella, he trains so much, it became an addiction to him. He became really addicted to training. See, if he couldn't train, he was crabby.
6	The progression over the 12 weeks was phenomenal.
62	Just in terms of physical fitness, feeling a bit healthier and fitter you know.
51	I haven't really tried, tested my weight but I have lost inches
61	It makes ya sleep better as well. I’ve been sleeping a lot better.
79	The best thing that you can do to combat arthritis is exercise if only walking one time a day.
24	Erm, physical activity…it takes away the aches and pains in your body, you're not as stiff.
9	It's very, very important, but mental health……it does relate to all the conditions, so if somebody has cancer or they have cardiac issues, you know, there is mental health involved in all of them.
68	I felt better when I exercised that day and the next day, I felt much better.
60	I enjoy it. It lifts your mood a bit…
26	The exercise I think it releases endorphins in the brain, to make you feel better about yourself.
44	You see when you do these exercises, it releases these endorphins and it makes you feel happier. You feel brighter.
75	It just means good for your bones and your brain…it stimulates your mind and you get a laugh from it.
78	And it sort of exercises your mind and your body at the same time
